# Design of highly perceptible dual-resonance all-dielectric metasurface colorimetric sensor via deep neural networks

**DOI:** 10.1038/s41598-022-12592-9

**Published:** 2022-05-20

**Authors:** Hyunwoo Son, Sun-Je Kim, Jongwoo Hong, Jangwoon Sung, Byoungho Lee

**Affiliations:** 1 Inter-University Semiconductor Research Center, School of Electrical and Computer Engineering, Seoul National University, Gwanakro 1, Gwanak-Gu, Seoul, 08826 Republic of Korea; 2 Department of Physics, Myongji University, Myongjiro 116, Namdong, Cheoin-Gu, Yongin, Gyeonggi-Do 17058 Republic of Korea

**Keywords:** Engineering, Nanoscience and technology, Optics and photonics

## Abstract

Colorimetric sensing, which provides effective detection of bio-molecular signals with one’s naked eye, is an exceptionally promising sensing technique in that it enables convenient detection and simplification of entire sensing system. Though colorimetric sensors based on all-dielectric nanostructures have potential to exhibit distinct color variations enabling manageable detection due to their trivial intrinsic loss, there is crucial limitation that the sensitivity to environmental changes lags behind their plasmonic counterparts because of relatively small region of near field-analyte interaction of the dielectric Mie-type resonator. To overcome this challenge, we proposed all-dielectric metasurface colorimetric sensor which exhibits dual-resonance in the visible region. Thereafter, we confirmed with simulation that, in the elaborately designed dual-Lorentzian-type spectra, highly perceptible variations of structural color were manifested even in minute change of peripheral refractive index. In addition to verifying physical effectiveness of the superior colorimetric sensing performance appearing in the dual-resonance type sensor, by combining advanced optimization technique utilizing deep neural networks, we attempted to maximize sensing performance while obtaining dramatic improvement of design efficiency. Through well-trained deep neural network that accurately simulates the input target spectrum, we numerically verified that designed colorimetric sensor shows a remarkable sensing resolution distinguishable up to change of refractive index of 0.0086.

## Introduction

Optics-based label-free biomolecular sensing platforms for identifying microscopic particles have attracted much attention due to various advantages in terms of real-time monitoring, lifetime, and operation bandwidth^1^. Over the last several decades, biomolecular sensing based upon nanophotonic transducer has made explosive advances in the progress of assays for various fields such as gases, solutions, nucleic acids, and proteins^2^. Its nano-scale footprints have contributed not only to miniaturization and reduction of production cost, but also to convenient integration into a lab-on-a-chip platform^3^. One of the most representative nanophotonic biosensors is based upon localized surface plasmon resonance utilizing light-metal interactions at the interface of metal particles and host dielectrics^4^. However, several crucial problems are inherent to these plasmonic sensors made of noble metals due to their intrinsic Ohmic losses and the nature of plasmon oscillations^5^. First of all, broadening of resonance linewidth caused by fast dephasing of surface plasmons acts as a bottleneck in improving read-out resolution and efficiency^6^. Furthermore, photo-thermal degradation of the analyte due to localized heating driven by high thermal conductivity of metals has been an obstacle for in-vivo sensing. To alleviate these limitations, high-index and low-loss all-dielectric nanostructures have been researched as an alternative.

In nanophotonics, all-dielectric nanostructures have become attractive solutions thanks to their capability of manipulating light in extraordinary and powerful manner. Among various organizations, all-dielectric metasurfaces, which are composed of periodic array of nanostructures, have been utilized for applications such as flat meta-optics^7–11^, highly saturated color generation^12–14^, and refractometric and colorimetric sensing with high quality factor^15,16^. In the context of sensing, in the recent years, all-dielectric metasurface sensors are becoming substitute for their plasmonic counterparts. Relatively lower intrinsic loss makes it durable to photo-thermal heating and negligible absorption-driven energy dissipation enables to strongly confine incident light to the near-field exciting sharp resonance. In addition, its fabrication can be processed using complementary metal-oxide-semiconductor (CMOS) compatible fabrication facilities. However, relatively low sensitivity to the environmental change has delayed its commercialization^6,15^. The reason can be inferred from the multipolar response of dielectric nano-resonator predicted by Mie theory which describes the resonant behavior of high-index subwavelength particles. Examining the electromagnetic (EM) field distribution of the excited modes within the dielectric, it is clear that the EM field is mostly concentrated inside the nanostructure. Therefore, unlike the plasmonic nanostructure that supports strong outer near-field by bounded free electron oscillation, dielectric nanostructure cannot react sensitively to the changes in surrounding environment. To address this issue, previous research has attempted to utilize spectrally interfered resonance features, such as bound states in the continuum or Fano resonance in order to enhance near-field and quality factor^17,18^. However, because of their excessive sensitivity to minute errors of geometric parameters, they involve problems about tolerance for the fabrication and measurement process causing reduction of production yields.

On the other hand, in the case of colorimetric sensing approach, a solution to the problems existent in the all-dielectric nanophotonic sensors can be presented from a different perspective than near-field optics. Colorimetry is one of the methodologies that can be applied to the nanophotonic sensing, which enables read-out with one’s naked eye by variations of color associated with interaction between an analyte and transducer. Nanoscale structural coloration arising from array of nanostructures has been utilized as the signal for the colorimetric detection^19–22^. Due to its straightforward detection process without the necessity of additional measurement devices, colorimetric sensing has been in the spotlight as attractive sensing platform that can glimpse feasibility of point-of-care diagnosis beyond the laboratory level. Since its sensing performance is determined by large color variation responding to minute environmental changes by nano-particle binding, its sensitivity can be enhanced by forming specific spectral lineshapes in the visible region that manifests structural color with easily perceptible color changes. Therefore, through exquisitely designing structures of metasurface which provides extraordinary degrees of freedom for optical manipulation, if reflected optical response that accurately simulates spectral lineshapes optimized for color difference maximization can be implemented, it can be a fresh approach to solve the above-mentioned issues existent in the all-dielectric nanophotonic sensors. Furthermore, as a means to achieve this, advanced parameter optimization techniques via data-driven machine learning can be a highly efficient design method where trained deep neural network (DNN) supports design process.

In recent years, inverse design techniques incorporating deep learning have emerged in the field of nano-optics for various applications such as accurate structural color design, broadband absorber, optical filter, and optical data storage device^23–26^. The traditional design method, finding geometric parameters that satisfies the desired optical response through iterative full-field EM simulations, should be processed by traversing vast design space demanding enormous computational cost as the degree of freedom for multi-dimensional representation increases. It is highly time-consuming as well as difficult to assure whether the obtained results are near-optimal or not. Whereas, the deep learning approach suggests optimal solutions within design space in an instant after going through a one-time investment of EM simulation to update the weights of DNN.

In this paper, we proposed novel design methods of highly perceptible colorimetric sensor by finding the optimal lineshape that causes a huge color variation even with little change of spectrum shape. As a result of the optimal spectrum finding process, it was a specific type of dual-resonance spectrum that could achieve our goal. Therefore, we utilized all-dielectric metasurface that can modulate distinct dual-resonance in the visible region, beyond the conventional spectroscopic sensing method using a single resonance. In order to search geometric parameters that accurately emulate elaborate specification of the target reflected spectrum expressing highly sensitive structural color, it was desirable to adopt bidirectional DNN incorporated inverse design approach. From the full wave simulations substituting geometric values acquired from bidirectional DNN, we achieved remarkable colorimetric sensing performance, resolving even little change in concentration of glucose solution.

## Results

### Design processes of dual-resonance metasurface colorimetric sensor

**Figure 1:**
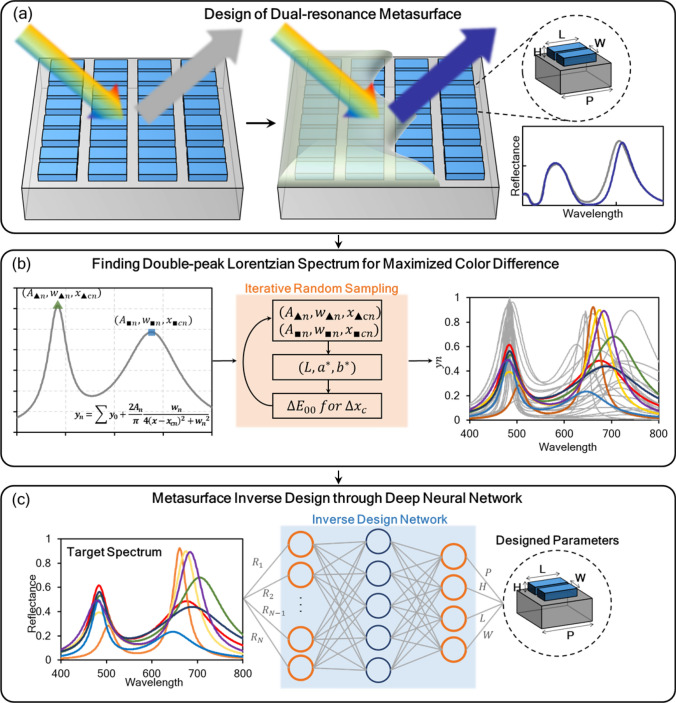
Overall design schematic diagram. (**a**) Design of double-bar structured metasurface introducing dual-resonance in visible wavelength and its application to colorimetric sensing. (**b**) Setting process of target spectrum through iterative random sampling of the sum of Lorentzian functions to specify the lineshape of dual-resonance. (**c**) Inverse design through deep neural network finding unit-cell parameters from the target spectra.

A schematic diagram for entire design processes is shown in Fig. 1. First, we found unit-cell geometry of metasurface that can excite distinct dual-resonance within visible region and analyzed its origin from EM field distributions and multipolar decomposition (Fig. 1a). The next step is to establish several target spectra that concretize specific form of dual-resonance achieving highly sensitive color change to minute spectral shift. Through an iterative random sampling method, we found highly perceptible spectra when calculated corresponding international commission on illustration LAB (CIELAB) coordinate values. Then, we finally summarized target spectra after considering the loss and dispersion of used material (Fig. 1b). Finally, by inserting these target spectra as an input of constructed bidirectional DNN which is trained through sufficient EM simulations, the detailed design of metasurface was searched (Fig. 1c). A detailed description of each design part will be dealt with in the following sub-sections.

### Design of dual-resonant metasurface

**Figure 2:**
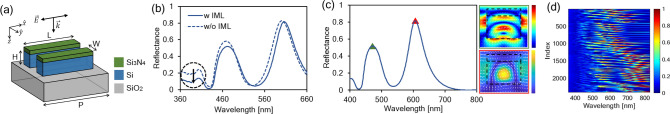
(**a**) A schematic illustration of the dual-resonance metasurface composed of parallel silicon rods covered by silicon nitride index matching capping layer onto them. (**b**) Background reflectance suppression effect obtained when 40 nm-thick index matching layer is applied. (**c**) Reflectance spectrum and electromagnetic field distributions of the corresponding resonance peaks (*P* = 290 nm, *H* = 160 nm, *L* = 210 nm, *W* = 50 nm). Electromagnetic field distributions are seen from the center of bar in *zx*-plane. At the peak of shorter wavelength (green square), arrows and color maps indicate the direction and amplitude of the electric field, respectively. In the case of longer wavelength (red square), color maps indicate the amplitude of magnetic field, but arrows correspond to the direction of electric field. (**d**) Reflectance map in the visible region calculated in design space when adjustable geometric parameters are set to *P, H, L,* and *W*.

As the first step, we designed metasurface that introduces dual-resonance. Analysis about the origin of superb colorimetric performance in dual-resonance spectrum with particular specifications will be dictated in the next section. We adopted double bar structure as a unit-cell of metasurface as described in Fig. 2a. Since the corresponding structure has been shown to introduce multiple resonant modes within a narrow wavelength range in the previous research, it can be noted to be prospective candidate for exciting dual-resonance^27^. We put 40 nm-thick silicon nitride layer on top of the double bar structured silicon layer. The dielectric constants of silicon and silicon nitride are taken from Palik and Philipp, respectively^28,29^. Stacked silicon nitride layer exhibiting refractive index value similar to the geometric mean of silicon and surrounding environment acts as an index matching layer suppressing high-order mode due to Fabry-Perot like resonance in short wavelengths^14^. The specific modulation effect of the stacking of SiN on reflectance and thickness optimization process is specified in the supplementary information S1. Accordingly, as shown in Fig. 2b, it is possible to attenuate background noise at short wavelengths, increasing similarity with the target spectrum and achieving more pronounced double peak spectrum overview. In the unit-cell structure, period (*P*), height (*H*), length (*L*), and width (*W*) were selected as variable parameters to be modulated. The center of the both bars are located at a distance of *P*/4 from the center of lattice. Figure 2c represents simulated representative dual-resonance spectrum in the visible region for *x*-polarized normal illumination when *P*=290 nm, *H*=160 nm, *L*=210 nm, and *W*=50 nm, respectively. Numerical simulations are conducted by finite-difference time-domain (FDTD) approach implemented in the Lumerical software package. To gain insight into the nature of each resonance, EM field distributions are numerically analyzed. Inset figure in Fig. 2c shows electric and magnetic field distribution at each resonance. At the resonance around the wavelength of 600 nm (bottom part of the inset), displacement current loop is formed with strong magnetic fields in the core of the resonator that corresponds to magnetic dipole (MD) mode. In addition, since this magnetic field formed by MD mode penetrates two parallel bars, interaction with their surroundings can occur more actively. These field distributions can assist the enhancement of spectroscopic sensitivity for bio-molecular detection. In the case of wavelength of 470 nm, electric near-field is strongly concentrated not only inside the resonator, but also on top of the substrate and between adjacent unit-cells (top part of the inset). It can be inferred that these field distributions arise from Mie lattice resonance^30,31^. Lattice resonance is collective resonance which occurs in periodic nano-array stemming from the radiative coupling of resonances of individual nano-resonator. It is enhanced near specific wavelength where diffraction order occurs, called Rayleigh anomaly (RA)^32,33^. At wavelength slightly higher than the RA, lattice resonance can occur and be reinforced as the coupling effect arises. In the proposed structure, corresponding RA wavelength is calculated by multiplying refractive index of substrate and period; *λ* = *nP* = 423.4 nm. Therefore, at the wavelength about 470 nm, by the radiative coupling around the RA wavelength, distinct reflection peak appears as the resonant mode introduced by hybridization of electric quadrupole and electric dipole moment is strengthened. Mode profile penetrating the top of the substrate serves to further strengthen coupling effect between adjacent unit-cells. Multipole decomposition results and detailed EM field distributions in each resonance are included in supplementary information S2. Meanwhile, in order to train DNN for an inverse design process, we constructed training sets from enough full-wave simulation results obtained by changing four structural variables (*P*, *H*, *L*, and *W*). Entire simulated reflectance spectra are shown as two-dimensional color map in Fig. 2d. Ranging from 245 to 400 nm for *P*, from 70 to 190 nm for *H*, from 170 to 290 nm for *L*, and from 40 to 150 nm for *W*, a total of 2,637 spectra were obtained. From the reflectance color map, we confirmed that dual-resonance can occur and be modulated within the design space of the proposed structure.

### Finding double-peak Lorentzian spectrum for maximized color difference

The next step is to find an optimized reflectance spectrum in the visible to maximize the color difference per refractive index unit (RIU) of the proposed dual-resonance metasurface colorimetric sensor. Prior to the spectrum finding process, we firstly needed to define a figure of merit (FoM) for colorimetric sensing. Previous studies about colorimetric sensing adopted the change in chromaticity per RIU as FoM caused by resonance shift^19,22^. However, as this metric does not reflect changes in saturation and brightness of color, it does not accurately correspond to color changes people perceive with naked eyes in reality. Therefore, we utilized CIEDE2000 (Δ*E*_00_) for quantitative and practical analysis of color difference, which has been developed in the field of color science. In order to resolve extant perceptual non-uniformity issue in CIELAB color space, Δ*E*_00_ is the latest color distance metric defined by refining CIE76 (*ΔE*) value which is a Euclidean distance between two colors in CIELAB space^34^. A detailed description for the CIELAB color space and the definition of Δ*E*_00_ are included in supplementary information S3.

To derive reflectance spectrum optimized for color difference detection, we used sum of multiple Lorentzian functions, one of the ideal symmetric spectral lineshape functions, which are most frequently observed ones in phenomena related to light radiation. In the case of asymmetric spectra such as Fano lineshape, which is frequently utilized in previous research to enhance sensitivity of nanophotonic sensor by reinforcing local field near the nanostructure, due to influence of the continuum state existent in entire region of spectrum, inevitable background noise exists from the perspective of colorimetry^18,35,36^. On the other hand, Lorentzian lineshape is more advantageous to enhance color purity and colorimetric sensing performance (Fig. S2a) because of lower background reflection. The Lorentzian function can similarly fit the dual-resonant reflectance spectrum appearing on the proposed metasurface. Therefore, the *n*th arbitrary spectrum for the target spectra to enter input of DNN is expressed by the following Lorentzian function.

1 $$ y_{n} = { }\mathop \sum \limits_{i} y_{0} + \frac{{2A_{i,n} }}{\pi }\frac{{w_{i,n} }}{{4\left( {x - x_{ci,n} } \right)^{2} + w_{n}^{2} }},{ }360 \le x \le 830 $$

In the above equation, ${{A}_{i,n},{w}_{i,n},\text{ and }x}_{ci,n}$ represent amplitude, linewidth, and wavelength of each resonance of the *n*th arbitrary spectrum, respectively. Using (1), random values of ${A,w,\mathrm{and }x}_{c}$ are iteratively generated. Then, for each candidate spectrum, we calculated the CIELAB coordinate value $({L}^{*},{a}^{*},{b}^{*})$ under the standard illuminant condition. From the calculated $({L}^{*},{a}^{*},{b}^{*})$, Δ*E*_00_/nm is calculated for slight spectral shift and only the candidates above a certain figure of Δ*E*_00_/nm were picked out. These random sampling processes are sufficiently repeated until a specific tendency was found in the selected lineshapes. Detailed procedures can be found in supplementary information S4.

**Figure 3:**
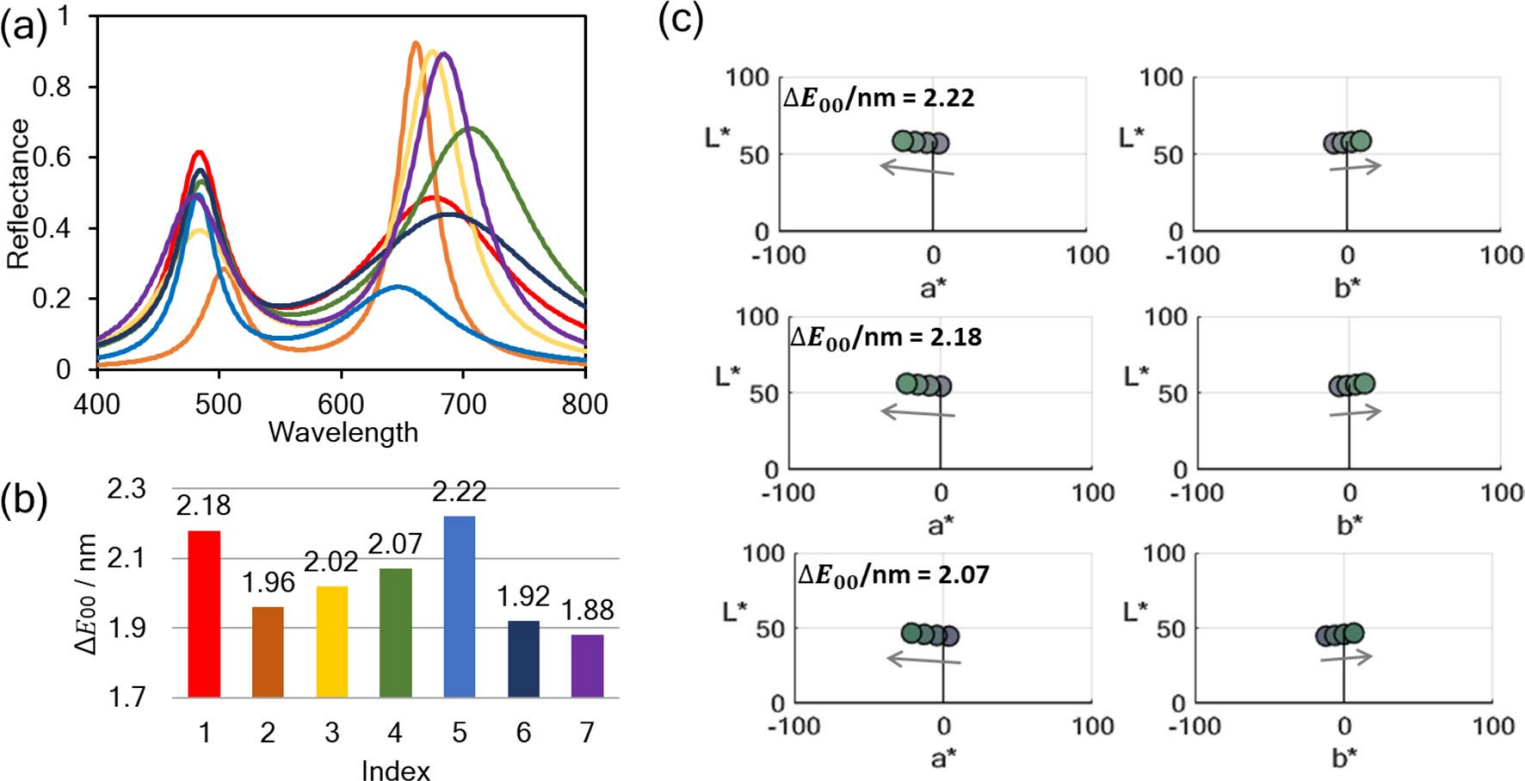
(**a**) Seven target spectra finally established in consideration of maximization of color difference per refractive index unit and visible loss of silicon. (**b**) Δ*E*_00_/RIU values calculated for each target spectrum. (**c**) For three target spectra with the highest Δ*E*_00_/RIU values, when the spectrum is laterally shifted by 5 nm, variations of colors are represented on two-dimensional section of CIELAB coordinate. The color inside the circle shows the reflected structural color calculated for each spectrum.

Figure 3a and b exhibit the finally selected seven target spectra and the corresponding Δ*E*_00_/nm values obtained from abovementioned processes after considering the optical loss in the visible area of silicon material. From Fig. S2c in supplementary information S4, it is apparent that these dual-resonance type target spectra show color difference that exceeds that of single resonance with extremely sharp line-width. In Fig. 2c, we selected the three of the target spectra with the highest Δ*E*_00_/nm, and each calculated reflected color shifting these spectra by 5 nm in each step (*i.e.* $${\Delta x}_{cn}$$=5 nm) is shown on two-dimensional section of the CIELAB coordinates to analyze principles of the massive color difference in these dual-resonance spectra. In all instances, it is discernible that the calculated colors are commonly located in neutral region where both $${a}^{*}$$ and $${b}^{*}$$ are close to zero, and $${L}^{*}$$ indicating lightness has a value of about 50. The reason why massive colorimetric performance is presented in these specific areas on CIELAB could be found out by introducing the concept of discrimination threshold ellipse^37^. Discrimination threshold ellipse is defined as an area in which the human eye cannot differentiate colors inside the same ellipse even if they are different. In other words, the smaller the ellipse size is, the easier it is to recognize even trivial color variations. According to the previous color perception experiments^38,39^, the region with the smallest ellipse locates is in the vicinity of the origin at the ($${a}^{*},{b}^{*})$$ coordinate and $${L}^{*}$$ between 40 and 60. These values correspond exactly to the area in which the calculated color of the target spectra is located as shown in Fig. 3c, thus verifying the cause of the exceptional color difference generated by dual-resonance spectrum. Additionally, further analysis can be made from the spectral lineshape itself of the target spectra. Examining the target spectra, firstly in the resonance occurring at longer wavelengths, since the peak point is commonly located between 650 and 700 nm, when the analyte is adsorbed on sensor causing spectral red-shifts, parts of the resonance deviate outside the visible region. Therefore, the ratio of red constituting the structural color decreases rapidly, inducing dramatic color variations. In the case of resonance at shorter wavelengths, the resonance wavelength is located in the blue-green area (*i.e.* $$\lambda $$= 460 ~ 510 nm) in order to place the constituted color on coordinates of CIELAB where the abovementioned ellipse has the smallest size when combined with the first resonance. As a result, in the target spectra with high FoM, the resonance wavelengths of both resonances are commonly located at particular spots.

### Bidirectional deep neural network: characterization and evaluation

**Figure 4:**
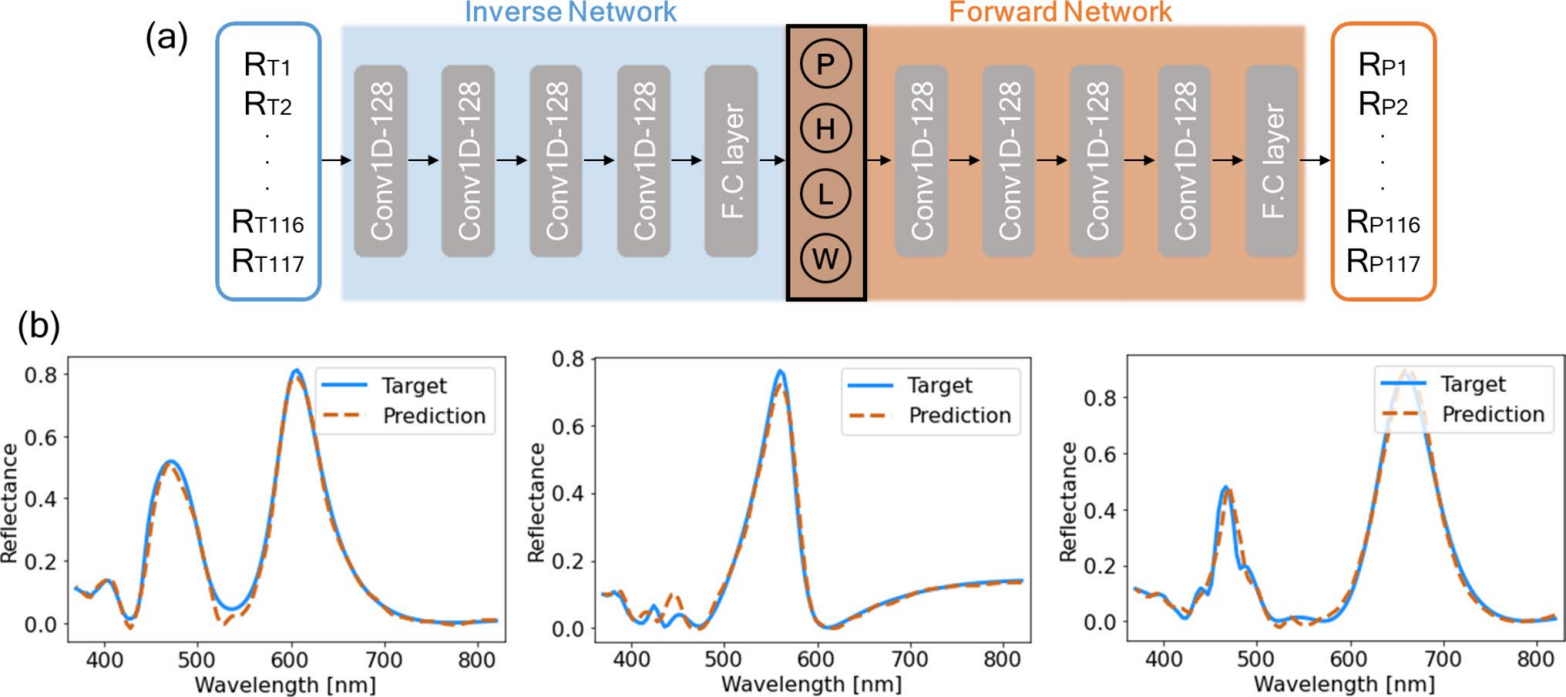
(**a**) Bidirectional deep neural network (DNN) architecture for inverse design of dual-resonant metasurface with input (output) layer of the target (predicted) spectrum divided into 117 wavelength points *R*_*Tn*_ (*R*_*Pn*_). Hidden layers are composed of four 1D-convolutional-layers (Conv1D-128), followed by fully connected layer (F.C layer). Inverse and forward DNN of same architecture are cascaded, and predicted normalized geometric parameters can be extracted from weight of the intermediate hidden layer where the two networks are replanted. (**b**) Comparison between target (*R*_*T*_) and predicted spectrum (*R*_*P*_) for different types of spectra obtained from test datasets showing that designed bidirectional DNN has sufficient forecasting accuracy.

We conducted multi-parameter optimization of the unit-cell of the proposed metasurface by training the DNN with thousands of full wave simulation data enabling more exquisite and time-efficient design process. We set up bidirectional network which cascaded inverse network at the input terminal of the pre-trained forward network. It is because the spectrum-geometry pairs have difficulty in constructing one-to-one-mapping (*i.e.* non-uniqueness problem) when establishing DNN architecture with general inverse network (direct prediction of geometric parameters from spectra)^23,40,41^. Architecture of the bidirectional network is shown in Fig. 4a. We put in the simulated reflectance for 117 wavelength points in the visible range between 360 and 830 nm (abbreviated by *R*_*TN*_) as an input to the inverse network. Inverse network predicts geometric parameters from *R*_*T*_, and the predicted reflectance *R*_*P*_ from these value by ensuing forward network becomes the output of the entire bidirectional network. Mean squared error (MSE) between *R*_*P*_ and *R*_*T*_ was set as loss function of the network, and normalized geometric parameters of the unit-cell can be found by extracting weight of the intermediate layer. As described in the first sub-section, a total of 2,637 spectrum-geometry pairs were obtained through EM simulations by setting (*P, H, L, W*) shown in Fig. 2a as geometric variables. These pairs were divided into 1,680 training sets and 945 validation and test sets. On the other hand, instead of dense layer, we utilized one-dimensional-convolutional layer (Conv-1D) to make DNN robust against overfitting, and improve regression accuracy^42^. As a result of trial-and-error for various hyperparameter conditions and the number of layers, optimized forward DNN architecture consists of 4-neuron input layer, 4 Conv-1D layers, followed by 256-neuron fully connected layer and 117-neuron output layer. Inverse network maintained equal architecture with the forward network but only changed order of input and output shape. The number of filters of each Conv-1D layer is 128 and kernel size is 3. A rectified linear unit (ReLU) activation function was applied to every end of layers. As a result of updating all of weights through Adam optimizer with learning rate of 0.001 and batch size of 16, highly accurate spectrum prediction capability could be obtained in the test set (MSE = $5.69\times {10}^{-4}$). For the bidirectional network which is constructed by connecting inverse network with pre-trained forward network, the same hyperparameter condition was substituted and trained over 5,000 epochs. Consequently, in the validation and test set, the losses were $1.2\times {10}^{-3}$, and $2.1\times {10}^{-3}$, respectively, implying that the network is well-converged. Figure 4b shows that the bidirectional network can almost accurately predict various reflected spectral shapes (*R*_*P*_) arising from the proposed metasurface obtained by FDTD simulations (*R*_*T*_). These results suggest that the designed bidirectional DNN can also serve as immensely time-efficient simulator in designing desired metasurface.

### Colorimetric sensing performance for optimized metasurface

**Figure 5:**
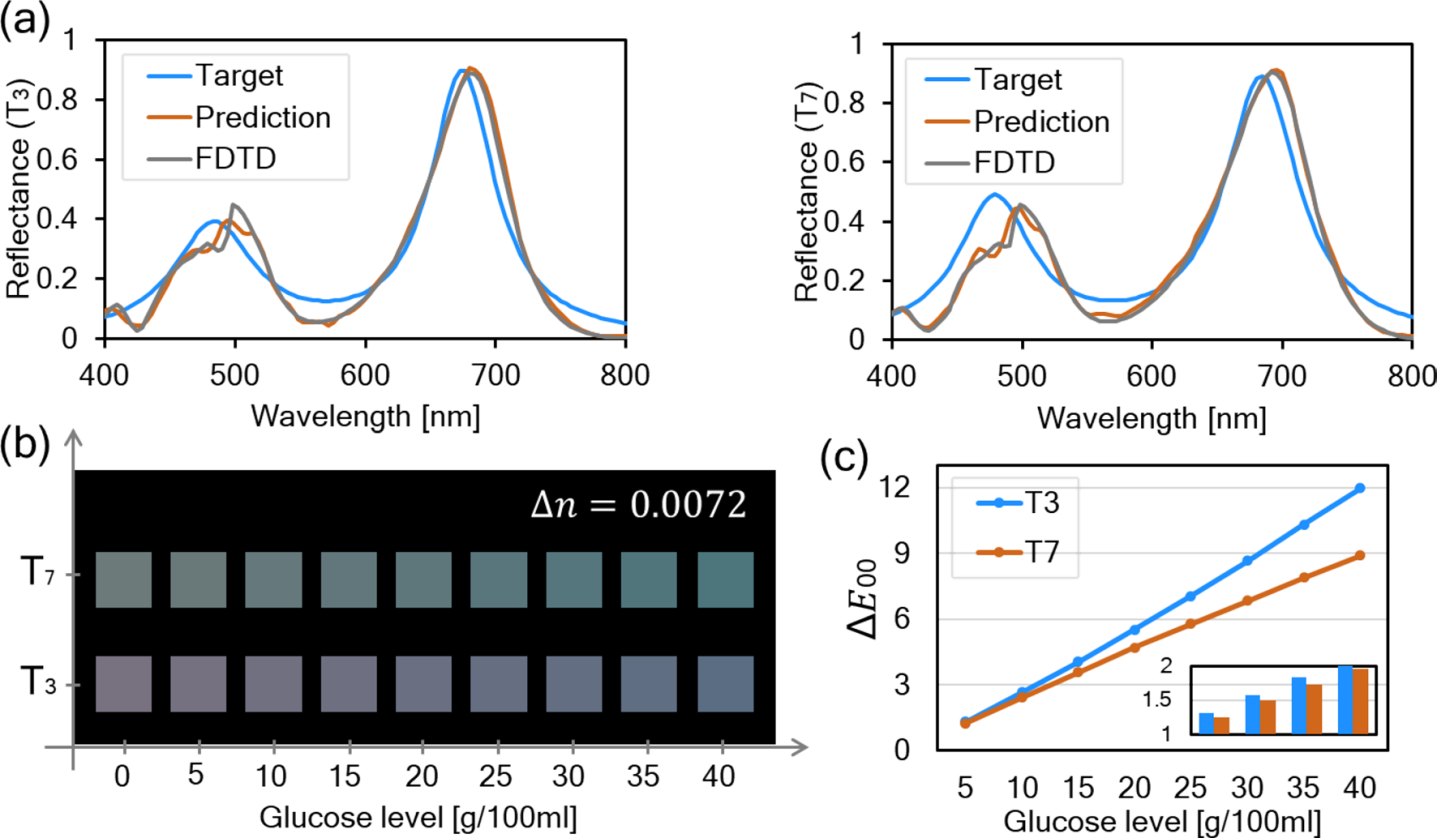
(**a**) Comparison between target spectral lineshape, predicted spectrum through DNN, and FDTD simulation results from the predicted geometric parameters according to the two target spectra showing the lowest loss from Table 1 (T_3_ and T_7_). (**b**) For T_3_ and T_7_, variations in reflected structural color appearing when glucose level is changed by 5 g/100 ml from 0 to 40. (**c**) Graph showing the calculated Δ*E*_00_ corresponding to the color difference shown in (**b**). Inset represents calculated Δ*E*_00_ when the glucose level is changed from 5 to 8 by 1 g/100 ml in order to determine resolution limit of the proposed colorimetric sensor.

Deploying abovementioned bidirectional DNN, we performed the inverse design of metasurface for 7 target spectra. Table 1 shows the geometric parameters the DNN predicted and the corresponding loss value. Among them, for the two spectra showing the lowest MSE (T3 and T7), we compared reflectance between target spectral lineshapes, FDTD simulation results obtained by substituting predicted geometric parameters, and DNN predictions as indicated in Fig. 5a. Although there is a slight discrepancy compared to the target due to the constraints of the design space of the proposed metasurface structure, thanks to the sufficient fidelity of the DNN, we could obtain predicted reflectance that is almost consistent with the FDTD simulation results. Afterward, in order to evaluate colorimetric sensing performance for these two most adequate cases, we displayed the change in structural color reflected from the designed metasurface according to environmental change. We selected glucose solution as target detection analyte which has been actively utilized in previous research about refractive index sensor because it well expresses the conditions for minute change in peripheral refractive index^20,43,44^. The refractive index per concentration of the glucose solution was calculated using the following equation^45^.

2 $$ n = n_{w} + 0.00143C, $$

where *n*_*w*_ is the refractive index of water which is set to 1.33, and *C* is a concentration of glucose in g/100 ml. Figure 5b indicates the reflected structural color according to the variation of glucose level by 5g/100 ml. Each step corresponds to change in refractive index (Δ*n*) of about 0.0072. Anyone can easily observe with naked eye that designed colorimetric sensor indicates notable sensitivity in color variation even with very little environmental changes. In Fig. 5c, the aforementioned results are expressed as a quantitative graph through Δ*E*_00_ with respect to the glucose level. From almost linearly proportional correlations between Δ*E*_00_ and concentrations, calculated Δ*E*_00_/RIU, FoM of the proposed colorimetric sensor, reaches about 190.23 for T_3_ and 165.58 for T_7_. According to the color recognition experiments conducted in Ref. ^46^, the minimum Δ*E*_00_ for noticeable color difference is specified as 1.5, and appreciable difference can be felt in 3.0 and above. That is, in agreement with calculated Δ*E*_00_ in each glucose level, the concentration over 15g/100ml (Δ*n* = 0.0215) can be detected clearly through the proposed colorimetric sensor for both cases (Δ*E*_00_ is 4.09 for T_3_, and 3.56 for T_7_). Furthermore, we can estimate that resolution limit of the sensor from the inset of Fig. 5c. From the inset showing Δ*E*_00_ per 1g/100ml of glucose level change, minimum concentration in which Δ*E*_00_ exceeds 1.5 is about 6g/100 ml of glucose level (Δ*n* = 0.0086) in the case of T_3_ and 7g/100 ml (Δ*n* = 0.0100) in T7.

**Table 1:** Geometric parameters predicted by designed DNN and corresponding mean squared error.

Target	P	H	L	W	MSE
${\text{T}}_{1}$	363.61	129.52	190.19	107.64	0.0316
${\text{T}}_{2}$	354.49	152.17	234.30	60.94	0.0103
${\text{T}}_{3}$	339.25	141.42	210.83	85.14	0.0042
${\text{T}}_{4}$	328.06	140.54	219.44	100.98	0.0150
${\text{T}}_{5}$	371.50	107.55	214.06	64.38	0.0244
${\text{T}}_{6}$	359.08	148.38	214.59	84.30	0.0290
${\text{T}}_{7}$	337.99	137.76	208.34	97.67	0.0064

## Discussion

In this article, we proposed highly perceptible colorimetric sensor by introducing unprecedented approach utilizing dual-Lorentzian resonant metasurface made of high index dielectrics. It maximizes the color difference by proposing a specific spectral lineshape unlike ways of previous research that focused only on increasing sensitivity of single resonance in terms of spectroscopy or near-field optics. Afterwards, we analyzed the origin about remarkable sensitivity of dual-resonance spectra from the viewpoint of colorimetry as well as spectroscopy. Furthermore, as a means to form precise spectral lineshape of the target reflectance without error, by grafting deep learning inverse design approach, we successfully achieved our goal with an error within 0.005 for MSE. Consequently, the designed all-dielectric colorimetric sensor could detect diminutive change in refractive index of less than 0.01 with the naked eye by alluding to the change in the concentration of glucose solution. These results can be a stepping stone for the growth of all-dielectric nanophotonic colorimetric sensors, whose development have been delayed by constraint on sensitivity due to its own resonant EM field distributions, allowing us to leverage miscellaneous benefits of all-dielectric photonic devices. Though we used high-index silicon to induce distinct dual-resonance within narrow spectral regions and to design within a manufacturable boundaries, if one uses materials whose visible loss converges to near-zero such as titanium dioxide and silicon nitride so as to successfully induces narrower dual-resonance in visible range, it may be possible to achieve higher colorimetric sensitivity. Alternatively, we expect that more precise resonance engineering can be permitted by increasing degree of freedom of design through unusual method such as configuring the unit-cell as supercell structure. We envision that our results would push the resolution limit of bio-molecular sensing based upon nanophotonic transducers and further serve as a momentum to open up new horizon in the sensing methodology.

## Methods

### Design of dual-resonant metasurface

Design of the dual-resonant metasurface are conducted by finite-difference time-domain (FDTD) approach implemented in the Lumerical software package. The dielectric constants of silicon and silicon nitride are taken from Palik and Philipp, respectively. The reflectance spectra were numerically calculated with vertically incident light polarized in the direction of long axis of the unit-cell of metasurface. Changing four variable parameters mentioned in the main text, a total of 2637 visible reflectance spectra were obtained through full-wave simulations.

### Finding double-peak Lorentzian spectrum for maximized color difference

The process of finding the visible reflectance spectrum optimized for color difference enhancement was performed through custom MATLAB code, iterative random sampling algorithm. Firstly, an arbitrary spectrum was represented by the sum of 0 to 3 Lorentzian functions, which has 3 variables each (a total of 9 variables). Thereafter, the CIELAB coordinate value when such spectrum corresponds to the visible spectral region, and Δ*E*_00_ for the minute spectral shift were calculated successively. Constantly repeating the above procedure with randomly sampled 9 variables of the sum of Lorentzian functions, only spectra with Δ*E*_00_ above a certain reference values were picked out until a certain tendency is found among the selected spectra.

### Construction of bidirectional deep neural network

DNN models are constructed under the open-source machine learning framework of TensorFlow. After configuring forward DNN which predicts reflectance spectrum (117 points) from the geometric parameters (4 variables) of the metasurface, inverse DNN which has the same model structure with the forward DNN is cascaded at the input terminal of the forward DNN. Forward DNN consists of 5 hidden layers, 4 Conv-1D layers and following 1 fully connected layer. Each Conv-1D layer has 128 filters with the kernel size of 3. Fully connected layer has 256 neurons. Loss function was set to mean squared error between input target and output estimated reflectance. A ReLU activation function was applied to every end of layers. Training of DNN are implemented through Adam optimizer with learning rate of 0.001 and batch size of 16 during 5000 epochs.

## Supplementary Information

Supplementary Information.
