# Author Correction: Design of highly perceptible dual-resonance all-dielectric metasurface colorimetric sensor via deep neural networks

**DOI:** 10.1038/s41598-022-16502-x

**Published:** 2022-07-26

**Authors:** Hyunwoo Son, Sun‑Je Kim, Jongwoo Hong, Jangwoon Sung, Byoungho Lee

**Affiliations:** 1 Inter-University Semiconductor Research Center, School of Electrical and Computer Engineering, Seoul National University, Gwanakro 1, Gwanak-Gu, Seoul, 08826 Republic of Korea; 2 Department of Physics, Myongji University, Myongjiro 116, Namdong, Cheoin-Gu, Yongin, Gyeonggi-Do 17058 Republic of Korea

Correction to: *Scientific Reports* 10.1038/s41598-022-12592-9, published online 20 May 2022

The Acknowledgments section in the original version of this Article was omitted. The Acknowledgments section now reads:

“This work was supported by the National Research Foundation of Korea (NRF) grant funded by the Korea government (MSIT) (No. 2020R1A2B5B02002730)."

The original Article has been corrected.

