# Supplementary material for: Design of highly perceptible dual-resonance all-dielectric metasurface colorimetric sensor via deep neural networks
Source: Sci Rep. 2022 May 20;12:8512. doi: 10.1038/s41598-022-12592-9 (PMC9122971; doi:10.1038/s41598-022-12592-9)
Supplement: Supplementary file 1 — Supplementary Information. [file 41598_2022_12592_MOESM1_ESM.docx]

Supplementary Information

Design of Highly Perceptible Dual-resonance All-dielectric Metasurface Colorimetric Sensor via Deep Neural Networks

# Hyunwoo Son1, Sun-Je Kim2, Jongwoo Hong1, Jangwoon Sung1, and Byoungho Lee1,*

1Inter-University Semiconductor Research Center and School of Electrical and Computer Engineering, Seoul National University, Gwanakro 1, Gwanak-Gu, Seoul 08826, Republic of Korea

2Department of Physics, Myongji University, Myongjiro 116, Namdong, Cheoin-Gu, Yongin, Gyeonggi-Do 17058, Republic of Korea

*Corresponding author: byoungho@snu.ac.kr

**S1. Influence of Silicon Nitride index matching layer**

**
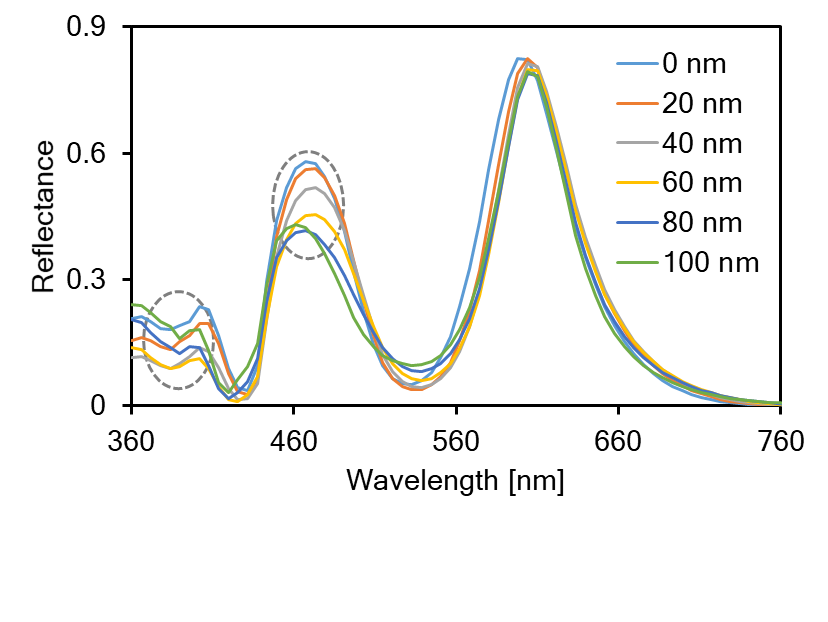
**

Figure S1. Reflectance spectra obtained by changing the thickness of the SiN index matching layer from 0 to 100 nm. Dotted ellipses represent sections where the spectrum is most affected by the thickness of the SiN layer.

In order to suppress background noise by high-order mode in short wavelengths, thereby increasing similarity with the target spectrum and achieving more pronounced double peak spectrum overview, silicon nitride (SiN) capping layer is stacked on top of the double bar structured silicon layer. Stacking this matching layer has the obvious advantage of reducing background noise in unwanted wavelengths, but comes at the inevitable cost of peak amplitude. However, looking at the lineshape of the 7 target spectra presented in Fig. 3. (a) of the main script, it is evident that the peak amplitude at the first resonance does not necessarily have to be high. Because they show commonly low peak amplitude values less than 0.6. That is why utilizing the SiN index matching layer is more advantageous in reaching our design goals. Through full-wave simulation results for various SiN thicknesses, it was confirmed that the optimal results were obtained when the thickness was 40 nm. The reflectance spectra obtained by changing the thickness from 0 to 100 nm are shown in the Fig. S2. Examining two areas highlighted by dotted ellipses, the background noise at short wavelengths and the first peak region, the background noise is best suppressed when the thickness of SiN is 40 nm and 60 nm. However, at the thickness of 60 nm, first peak amplitude is sacrificed more than that of 40 nm. Therefore, in this manuscript, a 40 nm SiN capping layer is assumed as an optimized thickness.

**S2. Multipole decomposition results and electromagnetic field distributions of dual-resonance metasurface**


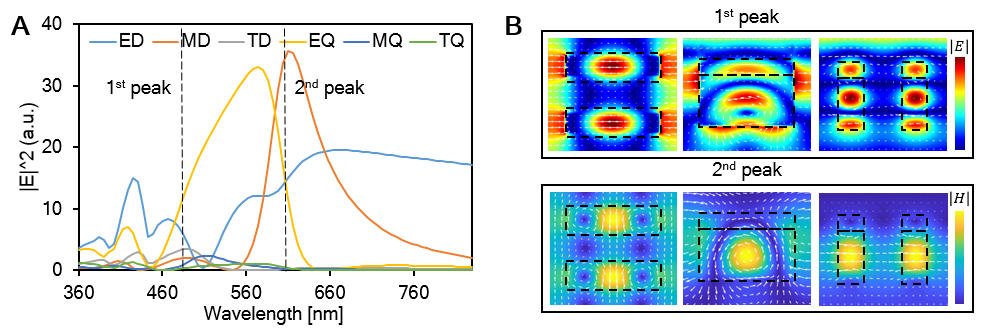


Figure S2. (A) Multipole decomposition results of dual-resonance metasurface. (B) Electric field distributions in each resonance peak. In the case of the 1st peak, electric field distribution is shown, and the 2nd peak is magnetic. All arrows indicate the direction of electric fields, but only the 2nd peak on *yz* plane is magnetic fields. In both peaks, in order from the left, the fields are calculated from the cross-sections cut into *xy*, *zx*, and *yz* plane, respectively.

For an in-depth analysis of the nature of two resonances, we performed multipole decomposition for the metasurface shown in Fig. 2 (A) with reference to the formulae in the previous study^1^. Figure S1. (A) represents the far-field intensities ($\left| E \right|^{2}$) induced by each multipole moment expressed up to the 2^nd^ order terms. At the first resonance peak indicated by dotted line in Fig. S1. (A), electric quadrupole moment is most dominant, and electric dipole made up second portion. Examining the corresponding electric field distribution as shown in Fig. S1. (B), at the field in *zx* section, upper side of each nano-bar shows the field distribution of electric dipole mode where the displacement currents (white arrows shown in Fig. S1. (B)) proceed aligned along the input polarization direction, while three anti-nodes opposite in sign observed in *zx* section indicate high-order mode dominated response (most dominantly electric quadrupole moment). It implies that it is forming a hybridized mode which is enhanced by Rayleigh anomaly (RA) induced lattice resonance^2–5^. As described in the first sub-section of the main text, we could observe that the resonant mode is concentrated even outside of the unit-cell (between adjacent unit-cells and the upper part of the substrate) due to the reinforced coupling effect between adjacent unit-cells by diffraction near the RA wavelength, inducing a distinct sharp resonance. In the case of the 2nd resonance, magnetic dipole mode is confirmed with displacement current loop in each nano-bar, and it could be verified from multipole moments at the wavelength of 600 nm. In addition, as can be seen in the *xy* plane and *yz* plane, induced magnetic field vector progresses penetrating on the two nano-bars, serving to higher spectroscopic sensitivity according to the changes in the surrounding environment.

**S3. Calculation method of CIELAB coordinate and CIEDE2000 for the reflectance**

As a figure of merit (FoM) of the designed colorimetric sensor, we utilized CIEDE2000 (Δ*E*_00_), as an indicator that numerically represents the color difference^6^. This value is defined on CIELAB coordinate. Because CIELAB is relatively perceptually-uniform color space, the color distance can be easily estimated with Euclidean measurement. We firstly computed the CIEXYZ coordinate values (*X, Y, Z*) and converted them to LAB values (*L, a, b*). (*X, Y, Z*) is calculated using the formulae below.

$$X=k\int S\left( \lambda\right)R\left( \lambda\right)\bar{x}\left( \lambda\right)d\lambda,$$

$$Y=k\int S\left( \lambda\right)R\left( \lambda\right)\bar{y}\left( \lambda\right)d\lambda, (S1)$$

$$Z=k\int S\left( \lambda\right)R\left( \lambda\right)\bar{z}\left( \lambda\right)d\lambda.$$

$(\bar{x},\bar{y},\bar{z})$ are color matching functions, which are described for associating colors with tristimulus values. $R\left( \lambda\right)$ is spectral reflectance calculated from FDTD simulation. $S\left( \lambda\right)$ is spectral power distribution of the illuminant. We used standard illuminant D65 as a data of $S\left( \lambda\right)$ for the following computations. Scaling factor *k* is set to $\frac{100}{\int\bar{y}\left( \lambda\right)S\left( \lambda\right)}$. We converted (*X, Y, Z*) obtained by S1 to (*L, a, b*) through following equations.

$$L^{*}=116f\left( \frac{Y}{Y_{n}} \right)-16,$$

$$a^{*}=500\left( f\left( \frac{X}{X_{n}} \right)-f\left( \frac{Y}{Y_{n}} \right) \right),$$

$$b^{*}=200\left( f\left( \frac{Y}{Y_{n}} \right)-f\left( \frac{Z}{Z_{n}} \right) \right),$$

$$f\left( t \right)=\left\{ \begin{aligned} \sqrt[3]{t}, &t>0.008856 \\ \frac{t}{0.128419}+\frac{4}{29}, &otherwise. \end{aligned} \left( S2 \right) \right.$$

($X_{n},Y_{n},Z_{n})$ is set to (95.049, 100, 108.884) for the illuminant D65. Substituting the reflectance of the designed metasurface obtained from FDTD simulations, we calculated the CIELAB values of the structural color for each spectrum.

In the field of color science, in order to quantify the distance between two colors, they defined a metric to represent the numerical color difference, and have developed it by correcting the pre-defined metrics. Originally defined formula, named $\Delta E$, is calculated by simple Euclidean distance between two colors $\left( L_{1}, a_{1}, b_{1} \right)$ and $\left( L_{2}, a_{2}, b_{2} \right)$. However, since the perceptual non-uniformity still remains in the CIELAB space, the calculated color distance does not accurately reflect the color difference perceived by human’s eye. It can be confirmed from the discrimination ellipses in the CIELAB mentioned in the main text showing non-isotropic shape^7–9^. In order to address this issue, by multiplying specific weighting factors so that the discrimination ellipse cluster on CIELAB shows an isotropic arrangement, modernized definitions have been defined over the years^10^. Among these, CIEDE2000 is the newest color distance metric which is well-coincided with human perceived color. It introduced several weighting and correction factors in lightness (*L*), chroma (*C*), and hue (*H*) which are defined by transforming CIELAB value to polar coordinate:

$${\Delta E}_{00}^{*}=\sqrt{{(\frac{\Delta L^{'}}{k_{L}S_{L}})}^{2}+{(\frac{\Delta C^{'}}{k_{C}S_{C}})}^{2}+{(\frac{\Delta H^{'}}{k_{H}S_{H}})}^{2}+R_{T}\frac{\Delta C^{'}}{k_{C}S_{C}}\frac{\Delta H^{'}}{k_{H}S_{H}}}. (S3)$$

Compensation for lightness, chroma, and hue is addressed by introducing *S*. $R_{T}$ is multiplied for correcting discrimination ellipse rotation in blue region. Primed values represent the compensation for neutral colors. Detailed calculation process for correcting factors is given in Ref. 6.

We utilized the Δ*E*_00_ per refractive index unit (Δ*E*_00_/RIU) as FoM of the proposed metasurface colorimetric sensor.

**S4. Characterization and comparison of optimal spectral line-shape for colorimetric sensing**


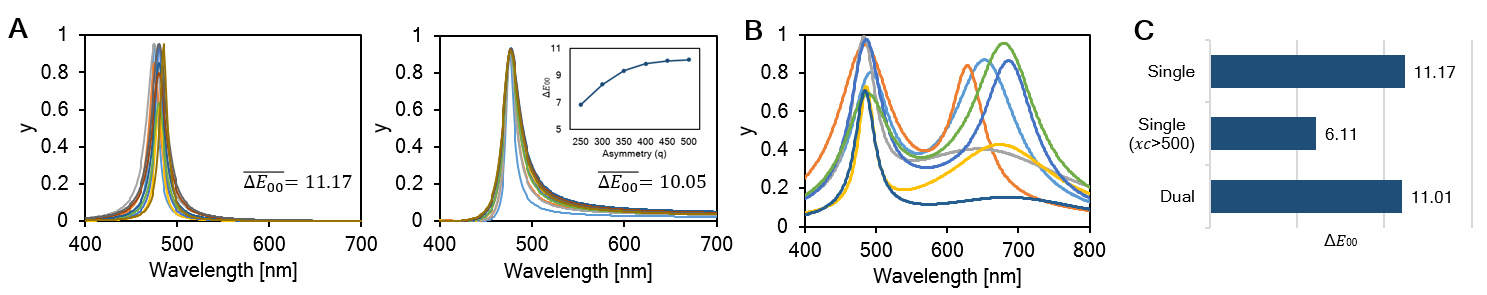


Figure S3. (A) Comparison of averaged Δ*E*_00_ between Lorentzian and Fano lineshapes. Inset in the Fano case indicates the correlation between line-shape asymmetry and Δ*E*_00_. (B) Eight line-shapes with the highest Δ*E*_00_ when randomly sample the sum of Lorentzian functions from zero to three. (C) Δ*E*_00_ mean of the 10 spectra with the highest color difference in each case of single Lorentzian function, single Lorentzian function with center wavelength ($x_{c}$) over 500, and double Lorentzian function.

In this section, we will verify that the most suitable spectral line-shape for maximizing color difference is a symmetric dual-resonance type and then give a detailed account for the process of finding optimized resonance linewidth, peak amplitude, and wavelength.

In the context of nanophotonics-based bio-molecular sensing, unusual resonant scattering phenomena such as Fano resonance or bound state in the continuum are frequently applied to increase sensitivity by strengthening near-field concentration^11–13^. Such resonant phenomenon caused by the interference between discrete and continuum state is usually accompanied by asymmetric spectral lineshape. However, from the viewpoint of colorimetric sensing, such asymmetric line-shape may act as an obstacle in sensitivity of color change. To dig into this issue, comparative analysis between the asymmetric Fano type and Lorentzian type representing symmetric lineshape is carried out. The equations below correspond to the Lorentzian and Breit-Wigner-Fano (BWF) functions used for comparative analysis where ($A,w,x_{c})$ are amplitude, line-width, and center position in S4, and $\left( H,x_{c},q,w \right)$ are height, center position, asymmetry parameter, and linewidth in S5.

$y=y_{0}+\frac{2A}{\pi}\frac{w}{4\left( x-x_{c} \right)^{2}+w^{2}} ,$ (S4)

$y=y_{0}+\frac{H{(1+\frac{x-x_{c}}{qw})}^{2}}{1+{(\frac{x-x_{c}}{w})}^{2}} .$ (S5)

After substituting abundant values for the variables of the two equations, we calculated CIELAB coordinate values assuming that they correspond to the spectrum in the visible region, and obtained Δ*E*_00_ for minute spectral shift of 5 nm. In both formulae, $x_{c}$ was composed of 60 points at the same interval between 400 and 700. In the case of the Lorentzian function, the number of sweeps of *w* was 40 and *A* was 60, thus Δ*E*_00_ was calculated for a total of 144,000 spectra. In BWF function, a total of 153,600 operations were performed with the number (*q, w, h*) of (80, 8, 4). Figure S2. (A) represents the 10 spectra with the highest Δ*E*_00_ values for each of two equations obtained after performing all of the aforementioned operations. As a result, the mean Δ*E*_00_ value is apparently higher in the symmetric Lorentzian function. In addition, Fano function with the highest Δ*E*_00_ is commonly near-symmetric similar to that of Lorentzian. When we calculated Δ*E*_00_ by changing *q* of the BWF equation, which is the parameter inversely proportional to asymmetry of Fano line-shape, it was confirmed that Δ*E*_00_ steeply decreased as the asymmetry increased. It can be interpreted that as the asymmetry increases, the background noise due to the continuum state that appears in the entire visible region adversely affects the color difference augmentation. Reflecting these results, we adopted the sum of multiple Lorentzian functions to mimic target spectrum optimized for colorimetric sensing. The next step is to randomly sample 9 variables of ($a_{n},w_{n},x_{cn})$ that compose the multiple Lorentzian functions between 0 and 3 and select the spectrum with the highest figure of merit (Δ*E*_00_/nm). After repeating this process millions of times, optimized spectra are shown in Fig. S2. (B). The spectra show a tendency that two peaks are concentrated in a specific wavelength range. From these results, we reasonably inferred that a specific dual-resonance spectrum exhibits higher color difference than that of single or triple peaks. After that, as a result of performing the above iterative random sampling again using sum of two Lorentzian functions, finally obtained target spectra is shown in Fig. 2. (A) of main text. Figure S2. (C) shows that FoM of these dual-resonance spectra is comparable to that of an extremely sharp single resonance, and when considering visible loss at shorter wavelengths of silicon to be used as well as difficulties in detecting sharp resonance in experimental phase, it implies that dual-resonance type spectrum can be much more reasonable option for the design of optical colorimetric sensor.

**References**

1. Savinov, V., Fedotov, V. A. & Zheludev, N. I. Toroidal dipolar excitation and macroscopic electromagnetic properties of metamaterials. *Phys. Rev. B - Condens. Matter Mater. Phys.* **89**, 205112 (2014).

2. Castellanos, G. W., Bai, P. & Gómez Rivas, J. Lattice resonances in dielectric metasurfaces. *J. Appl. Phys.* **125**, 213105 (2019).

3. Yang, J.-H. *et al.* Structural colors enabled by lattice resonance on silicon nitride metasurfaces. *ACS Nano* **14**, 5678–5685 (2020).

4. Rayleigh, Lord. On the dynamical theory of gratings. *Proc. R. Soc. London. Ser. A, Contain. Pap. a Math. Phys. Character* **79**, 399–416 (1907).

5. Kim, S.-J. *et al.* Reconfigurable all-dielectric Fano metasurfaces for strong full-space intensity modulation of visible light. *Nanoscale horizons* **5**, 1088–1095 (2020).

6. Luo, M. R., Cui, G. & Rigg, B. The development of the CIE 2000 colour-difference formula: CIEDE2000. *Color Res. Appl.* **26**, 340–350 (2001).

7. Luo, M. R. & Rigg, B. Chromaticity‐discrimination ellipses for surface colours. *Color Res. Appl.* **11**, 25–42 (1986).

8. Luo, M. R. & Rigg, B. BFD (l: c) colour‐difference formula Part 1 - Development of the formula. *J. Soc. Dye. Colour.* **103**, 86–94 (1987).

9. Oshima, S., Mochizuki, R., Lenz, R. & Chao, J. Modeling, Measuring, and Compensating Color Weak Vision. *IEEE Trans. Image Process.* **25**, 2587–2600 (2016).

10. Berns, R. S. *Billmeyer and Saltzman’s Principles of Color Technology*. (John Wiley & Sons, 2019).

11. Chong, K. E. *et al.* Refractive index sensing with Fano resonances in silicon oligomers. *Philos. Trans. R. Soc. A Math. Phys. Eng. Sci.* **375**, (2017).

12. Abujetas, D. R., Sáenz, J. J. & Sánchez-Gil, J. A. Narrow Fano resonances in Si nanocylinder metasurfaces: Refractive index sensing. *J. Appl. Phys.* **125**, 183103 (2019).

13. Su, W., Geng, Z., Qi, J. & Wu, H. Multi-Fano resonances in graphene coated all-dielectric metasurface for refractive index sensing with high figure of merits. *IEEE J. Sel. Top. Quantum Electron.* **27**, 1–6 (2020).
